# Gut fungal community composition analysis of myostatin mutant cattle prepared by CRISPR/Cas9

**DOI:** 10.3389/fvets.2022.1084945

**Published:** 2023-01-17

**Authors:** Li Gao, Song Wang, Miaomiao Yang, Lili Wang, Zhen Li, Lei Yang, Guangpeng Li, Tong Wen

**Affiliations:** ^1^Faculty of Biological Science and Technology, Baotou Teacher's College, Baotou, China; ^2^College of Life Science, Northeast Agricultural University, Harbin, China; ^3^State Key Laboratory of Reproductive Regulation and Breeding of Grassland Livestock, College of Life Science, Inner Mongolia University, Hohhot, China

**Keywords:** *MSTN* mutant, gut fungi, cattle, ITS, high-throughput sequencing, correlation

## Abstract

Myostatin (*MSTN*) regulates muscle development and body metabolism through a variety of pathways and is a core target gene for gene editing in livestock. Gut fungi constitute a small part of the gut microbiome and are important to host health and metabolism. The influence of *MSTN* mutations on bovine gut fungi remains unknown. In this study, Internal Transcribed Spacer (ITS) high-throughput sequencing was conducted to explore the composition of gut fungi in the *MSTN* mutant (MT) and wild-type (WT) cattle, and 5,861 operational taxonomic units (OTUs) were detected and classified into 16 phyla and 802 genera. The results of the alpha diversity analysis indicated that no notable divergence was displayed between the WT and MT cattle; however, significant differences were noticed in the composition of fungal communities. Eight phyla and 18 genera were detected. According to the prediction of fungal function, saprotroph fungi were significantly more abundant in the MT group. The correlation analysis between gut fungal and bacterial communities revealed that *MSTN* mutations directly changed the gut fungal composition and, at the same time, influenced some fungi and bacteria by indirectly regulating the interaction between microorganisms, which affected the host metabolism further. This study analyzed the role of *MSTN* mutations in regulating the host metabolism of intestinal fungi and provided a theoretical basis for the relationship between MSTN and gut fungi.

## Introduction

The gut microbiota consists of microbial cells, including bacteria, fungi, archaea, protozoa, and viruses, and their relevant genetic material exists in the gastrointestinal (GI) tract of the host (1). Although fungi constitute only a small part of the gut microbiome, they play a key role in the life activities of the host. Fungal colonization facilitates host resistance to pathogenic infections and modulates the immune system (2, 3). Gut fungi can also maintain the barrier function of the intestinal mucosal lining through synergistic, antagonistic, or symbiotic interactions with the gut bacteria (4, 5) and play important roles in host health and metabolism (6, 7). Fungi are the most effective microbial group for degrading lignocellulose and are the core microorganisms for excavating high-efficiency fiber-degrading enzymes (7). The diversity of fungal flora in the gut, including the gut bacteria, is related to the diet, age, physical condition, and environment of the host.

Myostatin (*MSTN*) is an important negative regulatory factor in skeletal muscle development (8). It is a core target gene for gene editing in livestock to improve meat quality with a lower fat percentage and a more lean meat yield. Luxi cattle with the *MSTN* gene mutations prepared by the Inner Mongolia University through the clustered regularly interspaced short palindromic repeats/CRISPR-associated protein 9 (CRISPR/CAS9) technology have more developed muscles and a higher lean meat yield (9). Previous studies had analyzed the changes in gut bacteria composition in *MSTN*-mutated cattle and found that the *MSTN* mutation affects the metabolism of the body by regulating the composition of gut bacteria, thereby affecting the growth traits of beef cattle (10, 11). However, there are no reports on the changes in *MSTN* mutant bovine gut fungi and their correlation with host metabolism.

In this study, the gut fungal compositions of the *MSTN* gene mutant (MT) and wild-type (WT) cattle were investigated, and the association between gut fungal composition and bacterial composition was analyzed. These findings are of great significance to the metabolism-regulating mechanisms of *MSTN* and the interactions between bacteria and fungi.

## Materials and methods

### Ethics statement

This study was performed according to animal care and ethics in China and was approved by the Animal Ethics and Welfare Committee (AEWC) of Inner Mongolia University and Baotou Teacher's College.

### Fecal sample collection, DNA extraction, ITS sequencing, and data analysis

The cattle used in the present study were presumably 24-month-old females raised as common beef cattle in a local ranch. Sample collection was performed as described in an earlier study (10). Total DNA was extracted from fecal samples using the QlAamp Fast DNA Stool Mini Kit (Qiagen, Germany) according to the manufacturer's instructions. The fungal Internal Transcribed Spacer 2 (ITS2) region was obtained by applying the universal primers ITS (ITS1F: 5'-ACTTGGTCATTTAGAGGAAGTAA-3' and ITS2R: 5'-GCTGCGTTCTTCATCGATGC-3') and sequenced using the Illumina NovaSeq platform. Trimmomatic v0.33 software was used to leach the raw reads acquired by sequencing. Next, Cutadapt 1.9.1 software was applied to acquire clean reads. Clean reads were spliced by overlapping, and the acquired spliced data were leached based on the range length of different regions. The final non-chimeric reads were obtained using the dada2 (12) method in the QIIME2 2020.6 (13) software to denoise and remove chimeric sequences. The resulting reads were then clustered into operational taxonomic units (OTUs) based on 97% sequence similarity. The diversity of the fungal flora within the samples was studied using the Shannon, Simpson, Chao1, and abundance-based coverage estimator (ACE) indices, and a non-metric multidimensional scaling (NMDS) analysis by binary Jaccard distances was performed to compare the differences in fungal composition in different samples. The linear discriminant analysis (LDA) effect size (LEfSe) analysis was conducted to identify functional biomarkers in WT and MT samples (LDA ≥4.0) (14). Fungal functions were predicted using the FUNGuild software, and Spearman's correlations were conducted to prioritize indicator species linking fungi and bacteria.

## Results

### Sequence statistics

**Figure 1:**
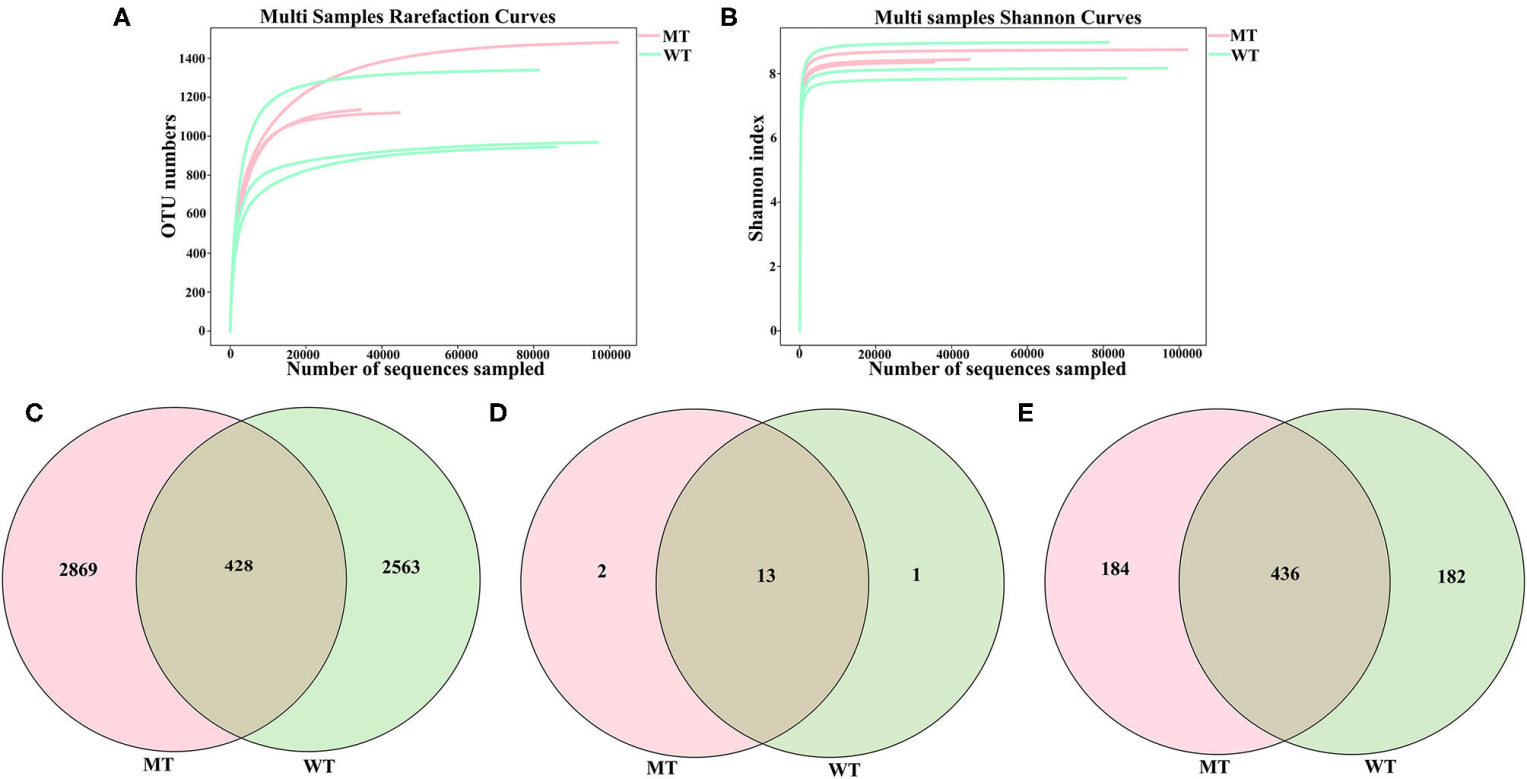
Feasibility analysis and operational taxonomic units (OTUs) distribution of the sequencing data. **(A)** Fungal rarefaction curves for all the samples; **(B)** Shannon curves for all the samples; **(C)** Gut fungal OTUs distribution in each group; **(D)** Gut fungal phylum distribution in each group; **(E)** Gut fungal genus distribution in each group.

A total of 492,564 (MT = 205,684, WT = 286,880) raw sequences of the ITS2 regions in the six sequenced samples (three MT and three WT cattle), with 35,429–102,161 (mean 74,452 ± 27,801) non-chimeric reads and 5,861 OTUs based on 97% sequence similarity, were obtained. These data were classified into 16 phyla, 64 classes, 159 orders, 372 families, and 802 genera. Both the Shannon diversity index and rarefaction curve tended to flatten, demonstrating that the depth and quantity of the sequencing met the requirements for further analysis (Figures 1A, B).

In total, the number of OTUs from the gut fungi shared by cattle in both the MT and WT groups was 428, while 2,869 and 2,563 OTUs existed only in the MT and WT cattle (Figure 1C), respectively. Thirteen phyla were shared by both MT and WT cattle, whereas two and one phyla existed only in MT and WT cattle, respectively (Figure 1D). At the genus level, 436 genera were shared by both MT and WT cattle, whereas 184 and 182 genera existed only in MT and WT cattle, respectively (Figure 1E).

### Analysis of alpha diversity and beta diversity

**Figure 2:**
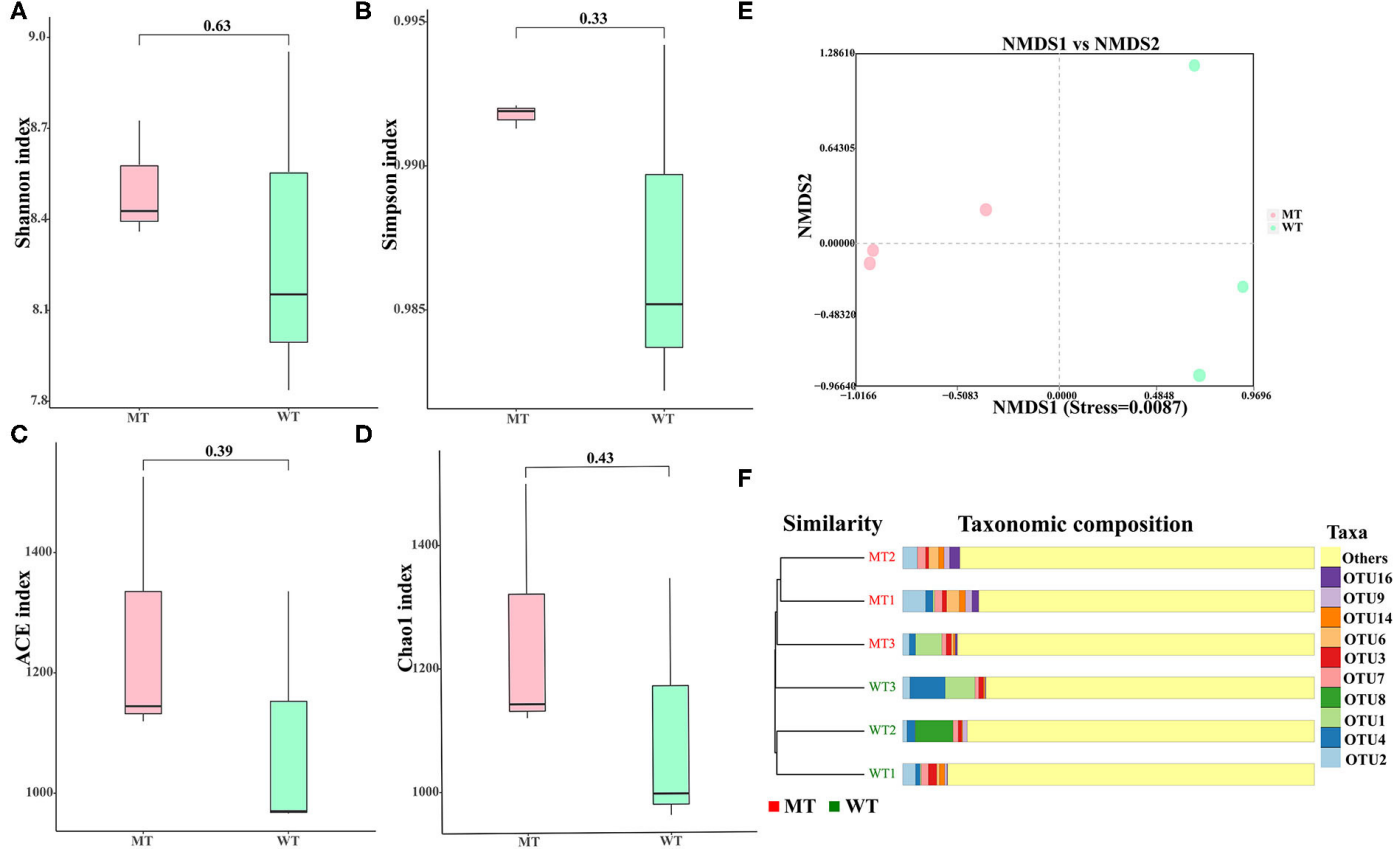
Gut fungal diversities of the wild-type (WT) and MSTN mutant (MT) cattle. **(A)** Shannon diversity; **(B)** Simpson indices; **(C)** abundance-based coverage estimator (ACE) index; **(D)** Chao1 diversity; **(E)** non-metric multidimensional scaling (NMDS) plots using Jaccard binary distance; **(F)** gut fungal clustering analysis.

No notable divergence was displayed in the Shannon (8.3326 ± 0.5766 vs. 8.5139 ± 0.2029, *P* = 0.65), Simpson (0.9871 ± 0.0063 vs. 0.9918 ± 0.0004, *P* = 0.33), ACE (1088.8220 ± 219.3710 vs. 1250.8343 ± 203.2833, *P* = 0.4), and Chao1 (1086.5587 ± 220.6933 vs. 1249.1780 ± 202.7929, *P* = 0.4) indices between the MT and WT cattle (*P* > 0.05) (Figures 2A–D), suggesting that alpha diversity had no notable divergence between the WT and MT cattle.

The divergence in the fungal composition between the WT and MT cattle was assessed using the NMDS based on binary Jaccard distances to generate scatter plots (Figure 2E) and the cluster tree bar plot (Figure 2F). The samples in the same group of cattle showed a significant clustering trend (stress < 0.05), suggesting that a significant difference was noticed in the composition of fungal communities between the WT and MT cattle.

### Holistic gut fungal community composition in the WT and MT cattle

**Figure 3:**
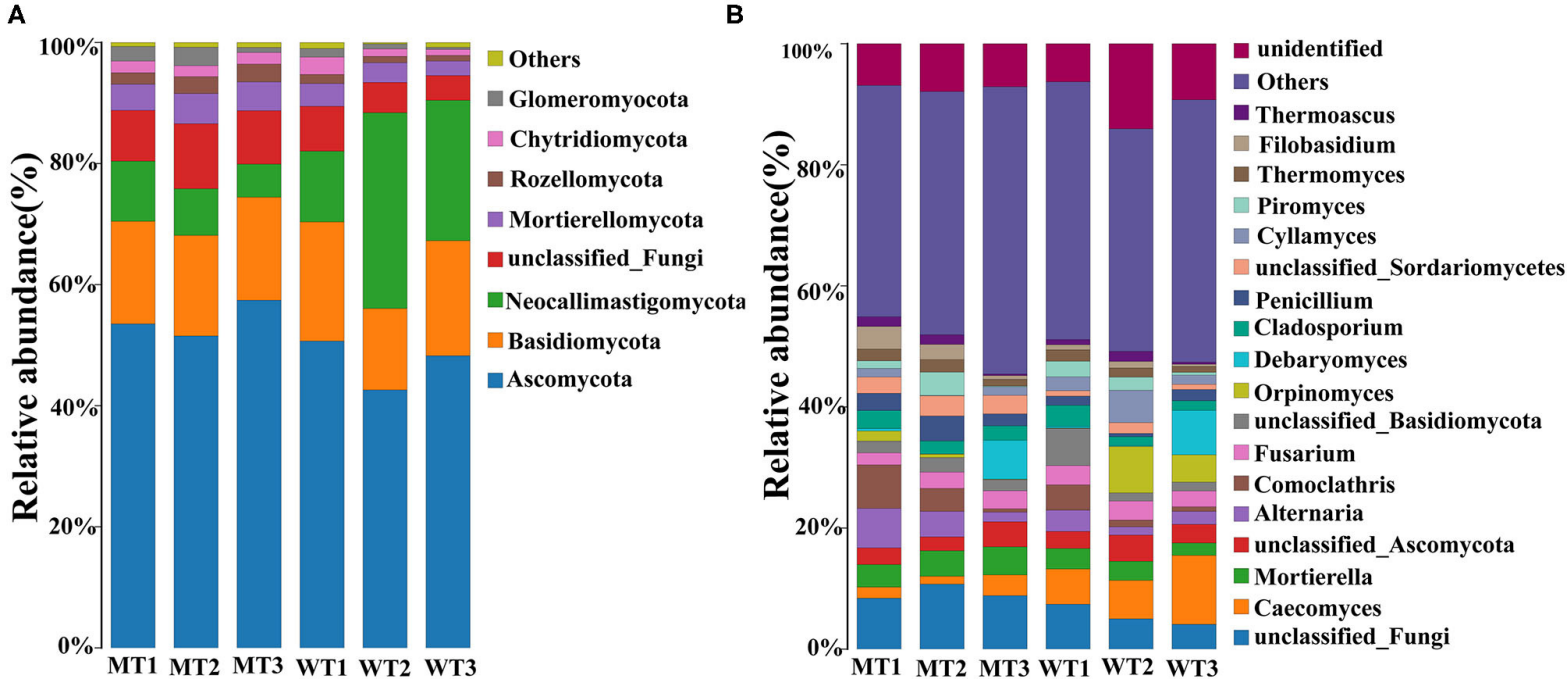
A bar chart showing the relative abundance of the fungi in MSTN mutant (MT) and wild-type (WT) cattle. **(A)** The relative abundance of the fungi at the phylum level; **(B)** The relative abundance of the fungi at the genus level.

The relative abundance of dominant fungi at the phylum and genus levels was analyzed, and visible changes in the fungal composition were noticed between the WT and MT cattle. Among the 16 phyla identified in the six fecal samples, the average relative abundance of eight phyla was >1% (Figure 3A), including Ascomycota (50.66%), Basidiomycota (17.12%), Neocallimastigomycota (15.05%), unclassified fungi (7.41%), Mortierellomycota (3.92%), Rozellomycota (1.82%), Chytridiomycota (1.79%), and Glomeromycota (1.48%). Three phyla were the most predominant in the MT group: Ascomycota (54.16%), Basidiomycota (16.86%), and unclassified fungi (9.31%); together they comprised 80.33% of the total fungal composition (Figure 3A). Moreover, Ascomycota (47.18%) was the most predominant fungal phylum in the WT group, followed by Neocallimastigomycota (22.40%) and Basidiomycota (17.38%), which comprised ~86.96% of all the fungal taxa in the WT group.

A total of 802 genera were detected, 18 of which had an average relative abundance of more than 1% (Figure 3B), including unclassified_fungi (7.41%), *Caecomyces* (5.02%), *Mortierella* (3.53%), unclassified_*Ascomycota* (3.24%), *Alternaria* (3.21%), *Comoclathris* (2.94%), *Fusarium* (2.76%), unclassified_*Basidiomycota* (2.53%), *Orpinomyces* (2.42%), *Debaryomyces* (2.42%), *Cladosporium* (2.35%), *Penicillium* (2.14%), unclassified_*Sordariomycetes* (2.11%), *Cyllamyces* (1.99%), *Piromyces* (1.74%), *Thermomyces* (1.59%), *Filobasidium* (1.52%), and *Thermoascus* (1.05%). Unclassified_fungi (9.31%) was the most predominant genus in the MT group, followed by *Mortierella* (4.20%) and *Alternaria* (4.10%), which together comprised ~50.74% of the total taxonomic groups identified. *Caecomyces* (7.85%), unclassified_fungi (5.50%), and *Orpinomyces* (4.08%) were the three most predominant genera in the WT group, comprising about 49.23% of the total taxonomic groups identified.

### Differences in gut fungal communities between the WT and MT cattle

**Figure 4:**
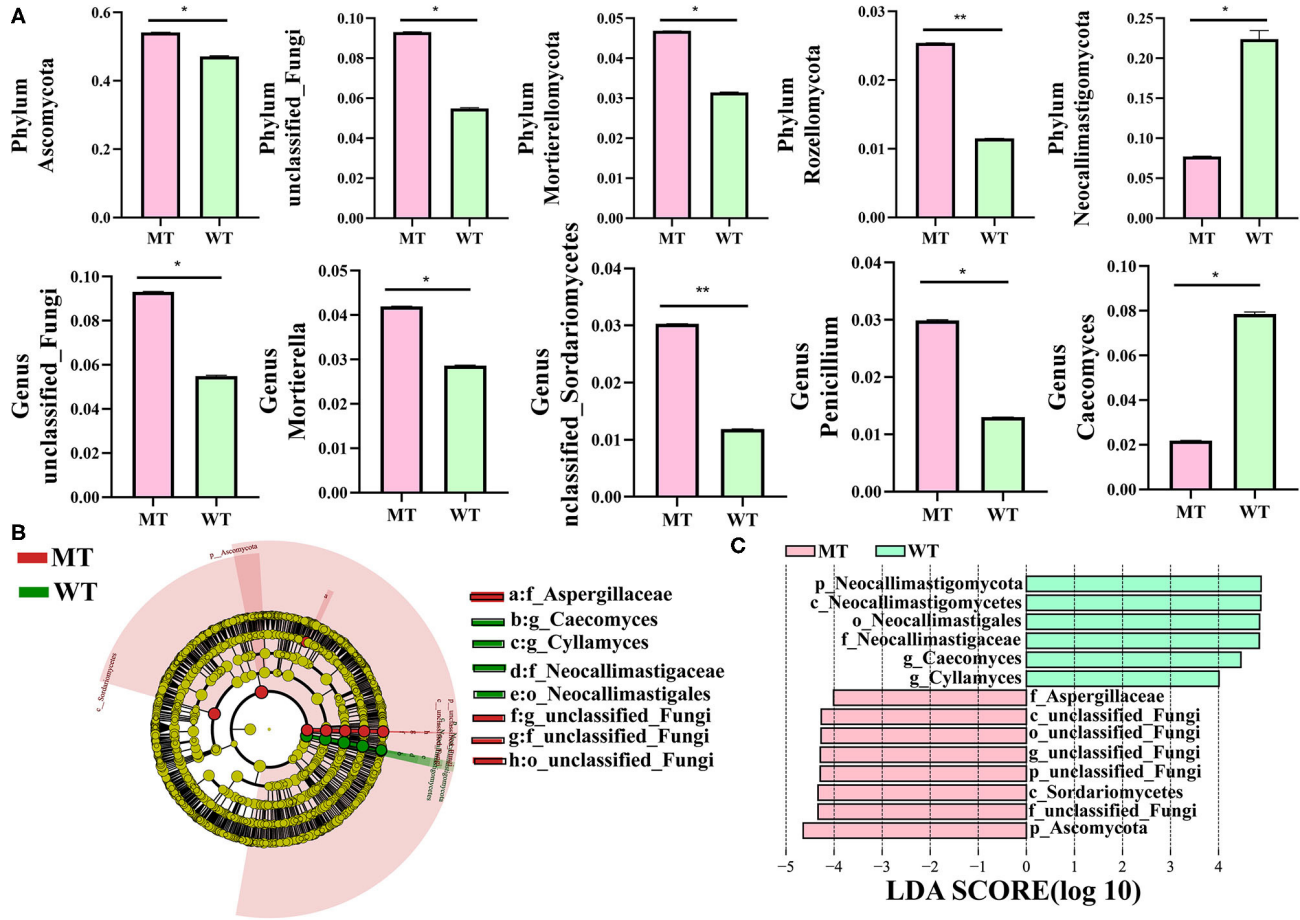
Comparisons of the gut fungal communities in MSTN mutant (MT) and wild-type (WT) cattle. **(A)** Metastatic analysis of those fungi whose relative abundance was more than 1%, and all the data stand for means ± SD. ^*^P < 0.05, ^**^P < 0.01. **(B)** Cladogram demonstrating the phylogenetic distribution of fungi related to the *MSTN* mutant. **(C)** Plot from linear discriminant analysis (LDA) effect size (LEfSe) analysis, linear discriminant analysis (LDA) scores >4.

The gut fungal composition in MT and WT cattle samples was identified further to analyze how *MSTN* mutations interfere with the gut fungal communities of the cattle, and a relative abundance of more than 1% was determined at different classification levels (Figure 4A). Ascomycota, unclassified_fungi, Mortierellomycota, and Rozellomycota were more dominant in the MT group compared to those in the WT group at the phylum level, whereas the relative abundance of Neocallimastigomycota was lower (*P* < 0.05) (Figure 4A and Supplementary Table S1). At the genus level, unclassified_fungi, *Mortierella*, unclassified_*Sordariomycetes*, and *Penicillium* were more predominant in the MT group than in the WT group, whereas *Caecomyces* was lower in the MT group compared to that in the WT group (*P* < 0.05) (Figure 4A and Supplementary Table S2). Considering that the discriminant analysis may not be able to detect the entire fungus, LEfSe plus LDA scores were employed to distinguish the specific fungi related to the *MSTN* mutant (Figures 4B, C). The results showed that unclassified_fungi was the most notable genus in the MT group compared to that in the WT group, whereas *Caecomyces* and *Cyllamyces* were dramatically more abundant in the WT group compared to those in the MT group.

### Fungal community functional prediction

**Figure 5:**
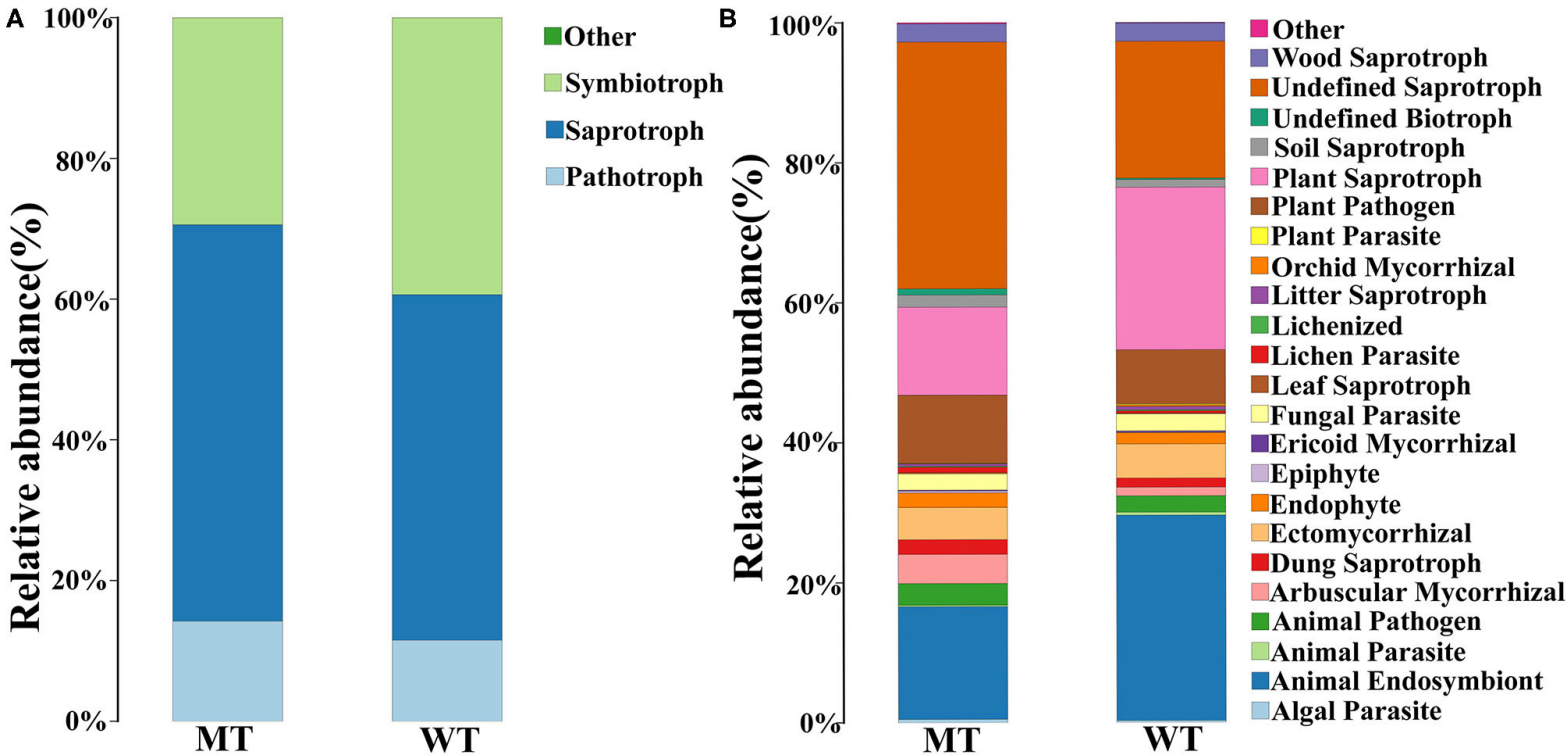
Fungal community functional prediction. **(A)** Function predicted in the trophic level. **(B)** Function predicted in the guild level.

The functions of fungi whose relative abundance was >1% were predicted by FUNGuild. According to the classification of nutrition, pathotroph fungi were more dominant in the MT group compared to those in the WT group but showed no significant differences (14.24 ± 2.74 vs. 11.52 ± 4.56%, *P* > 0.05), and saprotroph fungi were markedly more abundant in the MT group compared to those in the WT group(56.33 ± 2.62% vs. 49.10 ± 2.24%, *P* < 0.05), while the relative abundance of symbiotrophs was less in the MT group compared to that in the WT group, and no significant differences were observed (29.43 ± 0.87% vs. 39.38 ± 6.80%, *P* > 0.05) (Figure 5A).

When classified by guilds, the most important functions of both the MT and WT groups were undefined saprotrophs (27.41 ± 9.61%), animal endosymbionts (22.72 ± 9.24%), and plant saprotrophs (17.88 ± 6.84%). The most important functions in the MT group were undefined saprotrophs (27.41 ± 9.61%), animal endosymbionts (16.12 ± 3.12%), and plant saprotrophs (12.57 ± 2.64%). The most important functions in the WT group were animal endosymbionts (29.33 ± 8.54%), plant saprotrophs (23.20 ± 5.01%), and undefined saprotrophs (19.55 ± 5.66%), sequentially. The functions that were significantly different between the MT and WT groups were undefined saprotrophs and plant saprotrophs (*P* < 0.05) (Figure 5B).

### Correlation network analysis for the fungal communities

**Figure 6:**
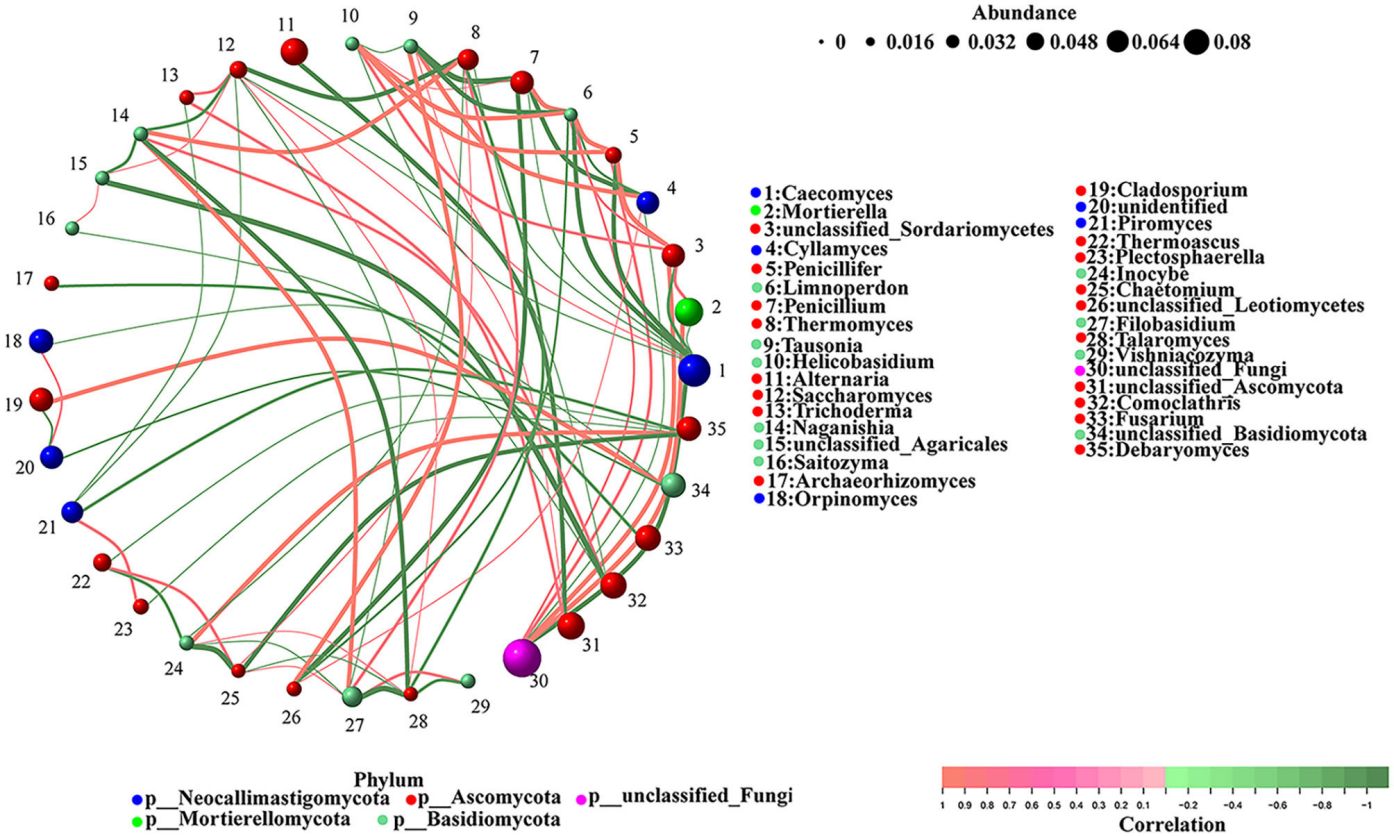
The correlations between different gut fungi. The color of the node indicates fungal taxa, and the relative abundance is represented by the weighted node size. Red lines show a positive correlation, while the green lines show a negative correlation.

The correlations between the different gut fungi were analyzed using the network analysis (Figure 6). The results indicated that unclassified_*Agaricales* was negatively correlated with *Comoclathris* (-1) and *Naganishia* (−0.8857) but positively correlated with *Saccharomyces* (0.8286). *Tausonia* was negatively correlated with *Penicillium* (-1) and positively correlated with *Caecomyces* (0.8286), *Cyllamyces* (0.9429), and unclassified_*Leotiomycetes* (0.9429). *Talaromyces* was negatively correlated with *Filobasidium* (-1), *Naganishia* (−0.9429), *Thermomyces* (−0.8857), and *Vishniacozyma* (−0.8857) and positively correlated with *Saccharomyces* (0.8286) and *Inocybe* (0.8286). *Chaetomium* was negatively correlated with *Inocybe* (-1), *Debaryomyces* (−0.9429), and *Talaromyces* (−0.8286) and positively correlated with *Thermomyces* (0.8286), *Filobasidium* (0.8286), and *Thermoascus* (0.8857). *Caecomyces* was negatively correlated with unclassified_fungi (−0.9429). *Penicillifer* was negatively correlated with *Caecomyces* (−0.9429) and positively correlated with unclassified_fungi (0.8857) and unclassified_*Sordariomycetes* (0.9429). *Alternaria* was negatively correlated with unclassified_*Ascomycota* (−0.9429). *Cyllamyces* was negatively correlated with *Penicillium* (−0.9429) and unclassified_fungi (−0.8286). Unclassified_*Leotiomycetes* was negatively correlated with *Penicillium* (−0.9429) and positively correlated with *Cyllamyces* (0.8286). *Saccharomyces* was negatively correlated with *Thermomyces* (−0.9429), *Comoclathris* (−0.8286), *Piromyces* (−0.8286), and *Filobasidium* (−0.8286) and positively correlated with unclassified_*Ascomycota* (0.8286). *Limnoperdon* was negatively correlated with *Caecomyces* (−0.9411), *Tausonia* (−0.9411), *Cyllamyces* (−0.8804), and unclassified_*Leotiomycetes* (−0.8804) and positively correlated with unclassified_fungi (0.8804), unclassified_*Sordariomycet* (0.8804), *Penicillium* (0.9411), *Penicillifer* (0.9411), and *Helicobasidium* (0.9411). Unclassified_*Sordariomycetes* was negatively correlated with *Caecomyces* (−0.8857) and positively correlated with *Mortierella* (0.8857) and unclassified_fungi (0.9429). *Archaeorhizomyces* was negatively correlated with *Fusarium* (−0.8857). *Piromyces* was negatively correlated with *Debaryomyces* (−0.8857). *Inocybe* was negatively correlated with *Thermoascus* (−0.8857), *Thermomyces* (−0.8286), and *Filobasidium* (−0.8286) and positively correlated with *Debaryomyces* (0.9429). *Naganishia* was negatively correlated with *Saccharomyces* (−0.8857) and positively correlated with *Comoclathris* (0.8857), *Thermomyces* (0.9429), and *Filobasidium* (0.9429). *Mortierella* was negatively correlated with *Caecomyces* (−0.8286) and positively correlated with unclassified_fungi (0.9429). *Penicillium* and *Thermomyces* were negatively correlated with *Caecomyces* (−0.8286). *Helicobasidium* was negatively correlated with *Caecomyces* (−0.8286) and *Tausonia* (−0.8286) and positively correlated with *Penicillium* (0.8286), unclassified_*Sordariomycetes* (0.8857), and *Penicillifer* (0.9429). *Saitozyma* was negatively correlated with *Comoclathris* (−0.8286) and positively correlated with unclassified_*Agaricales* (0.8286). *Orpinomyces* was negatively correlated with unclassified_*Basidiomycota* (−0.8286). *Thermoascus* and *Plectosphaerella* were negatively correlated with *Debaryomyces* (−0.8286). *Plectosphaerella* was positively correlated with *Piromyces* (0.8857). *Trichoderma* was negatively correlated with *Piromyces* (−0.8286) and positively correlated with unclassified_*Ascomycota* (0.8857) and *Saccharomyces* (0.8857). *Filobasidium* was positively correlated with *Thermomyces* (0.8857). *Vishniacozyma* was positively correlated with *Filobasidium* (0.8857), and *Cladosporium* was positively correlated with unclassified_*Basidiomycota* (0.9429).

### Relationship between gut bacterial and fungal communities

**Figure 7:**
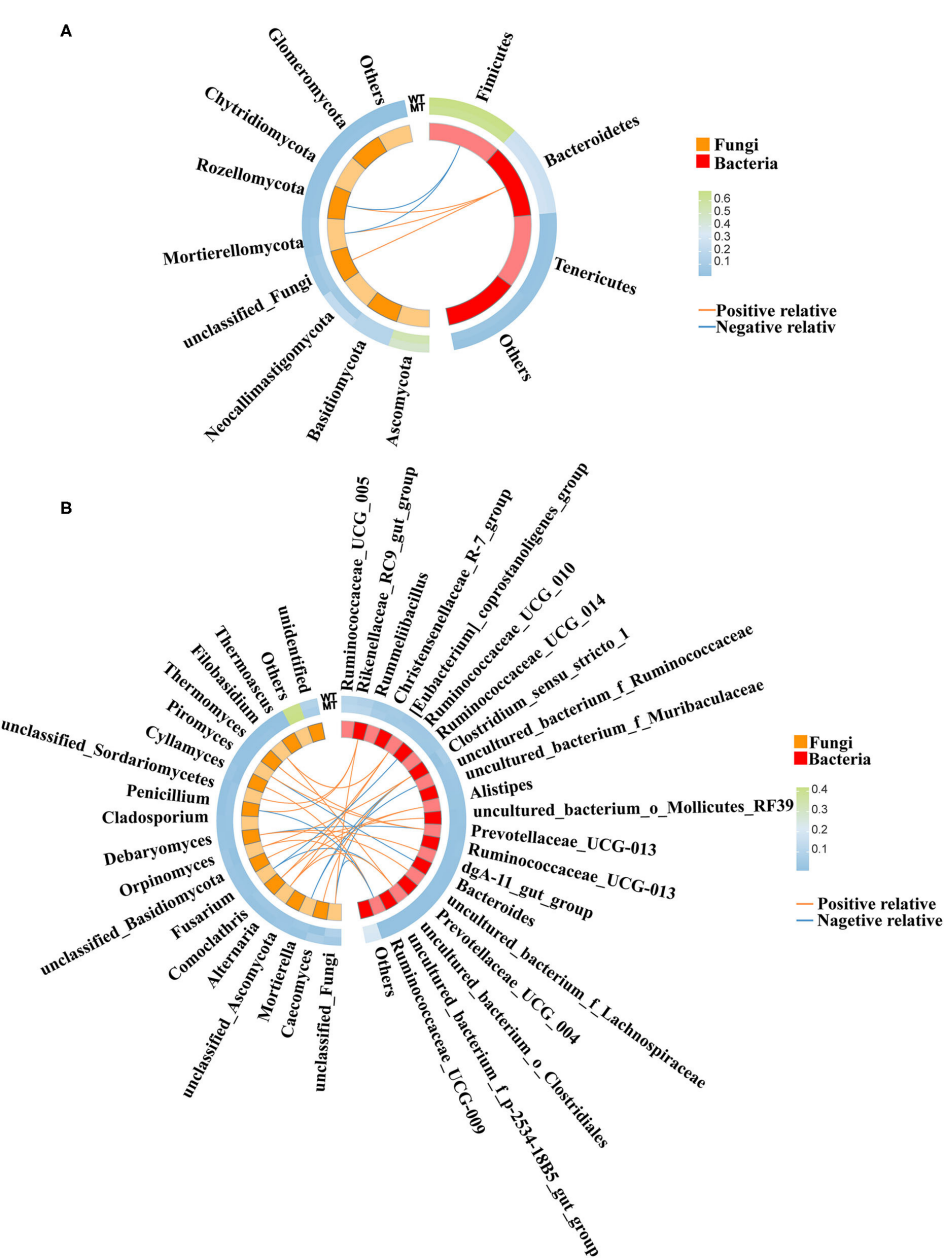
The correlation between the gut fungal communities and gut bacterial communities. **(A)** Correlation between gut fungal communities and gut bacterial communities at the phylum level. **(B)** Correlation between gut fungal communities and gut bacterial communities at the genus level.

The correlation between fungal and bacterial species was analyzed to distinguish the marker species linking fungi and bacteria. Spearman's correlation between multiple fungal species (>1% in each group) and bacterial species (>1% in each group) of the WT and MT cattle was determined. At the phylum level (Figure 7A), Mortierellomycota, Rozellomycota, and unclassified_fungi were positively correlated with *Bacteroidetes* (*P* < 0.05), and Rozellomycota and Mortierellomycota were negatively correlated with Firmicutes (*P* < 0.05).

At the genus level (Figure 7B), *Filobasidium* and *Alternaria* were positively correlated with uncultured_bacterium*_o_Clostridiales* (*P* < 0.05). Unclassified*_Basidiomycota* was positively correlated with uncultured_bacterium*_o_Mollicutes_RF39* (*P* < 0.05). *Comoclathris* and *Alternaria* were positively correlated with *Alistipes* (*P* < 0.05). *Debaryomyces* was positively correlated with uncultured_bacterium*_f_Lachnospiraceae* (*P* < 0.05). *Thermomyces* and *Piromyces* were positively correlated with *Prevotellaceae_UCG-003* (*P* < 0.05). *Caecomyces* and *Orpinomyces* were positively correlated with *Clostridium_sensu_stricto_1* (*P* < 0.05). *Alternaria, Comoclathris*, and *Filobasidium* were positively correlated with *[Eubacterium]_coprostanoligenes_group* (*P* < 0.05). *Cyllamyces* and *Orpinomyces* were positively correlated with *Ruminococcaceae_UCG-009* (*P* < 0.05). Unclassified_fungi, *Penicillium*, and unclassified*_Sordariomycetes* were positively correlated with *Rikenellaceae_RC9_gut_group (P* < 0.05). *Debaryomyces* was positively correlated with *dgA-11_gut_group* (*P* < 0.05). *Fusarium* was negatively correlated with *Ruminococcaceae_UCG-010* (*P* < 0.05). *Piromyces* was negatively correlated with *Bacteroides* (*P* < 0.05). *Penicillium* and unclassified_fungi were negatively correlated with *Ruminococcaceae_UCG-009* (*P* < 0.05), *Mortierella* was negatively correlated with uncultured_bacterium*_f_Ruminococcaceae* (*P* < 0.05), *Fusarium* was negatively correlated with uncultured_bacterium*_o_Clostridiales* (*P* < 0.05), *Mortierella* and unclassified_fungi were negatively correlated with *Clostridium_sensu_stricto_1* (*P* < 0.05), and *Debaryomyces* was negatively correlated with *Prevotellaceae_UCG-003* (*P* < 0.05).

## Discussion

*MSTN* is a negative regulator of muscle development in several species (15–20) and *MSTN* has been used as a target gene in animals for high-yield meat production. Many studies have been conducted to inhibit its expression through various strategies (21–23). Studies showed that *MSTN* can regulate multiple growth and metabolic pathways, such as muscle development, bone development (24), glucose metabolism (25, 26), and fat metabolism (27).

Fungi make up a relatively small proportion of the intestinal tract but are essential for the homeostasis of the intestinal flora (4, 5, 28). The gut flora participates in the maintenance of host health by influencing the metabolism and synthesis of some nutrients, vitamins, and hormones; by removing drugs and toxic metabolites; and contributing to the development and maturation of host immune cells to protect against pathogens (29, 30). Additionally, intestinal fungi are closely related to bacteria, and their stable antagonistic and mutually beneficial symbiosis is fundamental to the homeostatic balance of intestinal microorganisms. The present study previously detected bacterial communities in the *MSTN* mutant cattle and found that *MSTN* mutations affected the metabolism of the host by affecting the composition of the bacteria (10). However, knowledge of the characteristics of the gut fungal community composition in the *MSTN* mutation cattle is still limited. In the current study, ITS high-throughput sequencing was performed to investigate the fungal community composition of MT cattle. Furthermore, the relevance of the gut fungal community composition and bacterial composition was analyzed.

According to the alpha diversity analysis, no notable difference between the richness and diversity of the observed OTUs in the fecal samples of the WT and MT cattle was found, suggesting that the *MSTN* mutation had no remarkable effect on the gut fungal richness and diversity. The NMDS results based on the binary Jaccard analysis indicated that there were notable differences in the composition of the fungal community between the MT and WT cattle.

At the phylum level, Ascomycota, Basidiomycota, and Neocallimastigomycota were the predominant fungal phyla in the intestinal tracts of both the WT and MT cattle. This fact is consistent with previous studies on the dominant fungi of herbivores such as cattle (31), sheep (32), Tibetan piglets (33), and highland animals such as horses, yaks (34), and adult beach sheep (35). However, the relative abundance of Ascomycota and Neocallimastigomycota between the WT and MT cattle showed dramatic differences; Ascomycota was significantly higher in the *MSTN* mutation cattle compared to that in WT cattle, while Neocallimastigomycota was significantly lower in *MSTN* mutation cattle compared to that in WT cattle. Fungi at the phylum level in the digestive tract of ruminants help to depolymerize complex molecular structures such as lignocellulosic biomass, produce biogas and bioethanol, improve animal feed digestibility, and perform various other functions (36, 37). The direct feeding of anaerobic fungal cultures, such as Ascomycota, to ruminants can improve feed intake, milk quality, and milk production (38, 39). Members of Neocallimastigomycota usually live in the digestive tract of mammals (40, 41), and they are considered the most effective microbial group for degrading lignocellulose in the digestive tract of herbivores (42, 43). In the present study, the increased Ascomycota phylum and decreased Neocallimastigomycota phylum in *MSTN* mutation cattle may be one of the important reasons for the muscular development of *MSTN* gene-mutated cattle, but a detailed mechanism needs to be explored further.

At the genus level, unclassified_fungi, *Mortierella*, and *Alternaria* were the most predominant genera in the MT group, whereas *Caecomyces*, unclassified_fungi, and *Orpinomyces* were the most predominant genera present in the WT group. Unclassified_fungi, *Mortierella*, unclassified *Sordariomycetes*, and *Penicillium* were more predominant in the MT group compared to that in the WT group, while *Caecomyces* was lower in the MT group compared to that in the WT group (*P* < 0.05). All *Alternaria*, unclassified_*Sordariomycetes*, and *Penicillium* belong to the phylum Ascomycota, which are associated with improved animal production traits (38, 39). *Mortierella* is one of the most important fungi that produces polyunsaturated fatty acids (44, 45) and has various physiological functions, such as anti-inflammatory, anti-cancer, anti-coagulation, lowering blood lipids, and preventing cardiovascular diseases (46). *Caecomyces* and *Orpinomyces* belong to the phylum Neocallimastigomycota and function to decompose cellulose (40, 41). These results indicate that the mutation of *MSTN* in cattle not only improves the composition and proportion of microorganisms associated with meat quality production traits but also increases the content of beneficial fungi in the gut, which in turn regulates the metabolism of the body.

According to the prediction of fungal function, saprotroph fungi were significantly more abundant in the MT group than in the WT group. As a well-known active decomposer in the ecosystem, saprophytic fungi can decompose organic compounds such as animal and plant residues (47). They live on various dead organic substances, secrete digestive enzymes into these substances, and decompose them into small molecules such as glucose, which can be used by saprophytic fungi and other competitors (48). In this study, we found that the abundance of saprophytic fungi in the intestines of MT cattle was significantly increased compared to a similar abundance in the intestines of the WT cattle. Therefore, it was speculated that complex macromolecular organics can be degraded more efficiently and fully in MT cattle, allowing more nutrients to be absorbed and utilized by the host through the intestines, thus leading to enhanced growth and metabolism of the host.

Microbes inhabiting the intestinal tract can form symbiotic, synergistic, or antagonistic relationships that maintain the stability of the gut environment (49). For this reason, alterations in gut fungi and bacteria can influence the functions of other fungi and bacteria by regulating the interactions between microorganisms. The correlation analysis between the gut fungal and bacterial communities in the present study suggested that significant correlations were noticed between some conspicuously or not significantly changed fungi and bacteria influenced by the *MSTN* mutation, which may additionally influence global gut functions. These results suggested that the *MSTN* mutation changed the gut fungal and bacterial compositions directly while indirectly influencing some fungi and bacteria through the interaction of microorganisms, which affected the host metabolism further.

## Conclusion

The ITS gene high-throughput sequencing was conducted to explore the fungal community composition in the fecal samples of the *MSTN* mutant (MT) and wild-type (WT) cattle in this study. The gut fungi analysis revealed that the *MSTN* mutant had no effect on the richness and diversity of gut fungi. However, compared to wild type (WT) cattle, the composition of gut fungal community was changed in MSTN gene mutant (MT) cattle. According to the prediction of fungal function, the abundance of saprotroph fungi was significantly higher in the MT group compared to that in the WT group. The correlation analysis between the gut fungal and bacterial communities revealed that the *MSTN* mutation directly changed the gut fungal compositions and indirectly influenced some fungi and bacteria by regulating the interaction of microorganisms, which affect the host metabolism further. This study also analyzed the role of *MSTN* mutation in regulating the host metabolism of intestinal fungi and provided a theoretical basis for the relationship between the *MSTN* and gut fungi.

## Data availability statement

The datasets presented in this study can be found in online repositories. The names of the repository/repositories and accession number(s) can be found in the article/Supplementary material.

## Ethics statement

The animal study was reviewed and approved by Animal Ethics and Welfare Committee (AEWC) of Inner Mongolia University and Baotou Teachers College. Written informed consent was obtained from the owners for the participation of their animals in this study.

## Author contributions

LG and TW designed the research and wrote the manuscript. SW, LW, ZL, and MY collected the samples. LG and LY analyzed the data. GL and LY revised the manuscript. All authors contributed to the article and approved the submitted version.
